# Supplementary material for: SQUID-COMM: a Colossal Squid-inspired distributed communication framework for real-time multi-node aquaculture monitoring networks with adaptive bioluminescent signaling and neuromorphic edge intelligence
Source: Sci Rep. 2026 Jun 2;16:16982. doi: 10.1038/s41598-026-54545-6 (PMC13230730; doi:10.1038/s41598-026-54545-6)
Supplement: Supplementary file 1 — Supplementary Material 1 [file 41598_2026_54545_MOESM1_ESM.docx]

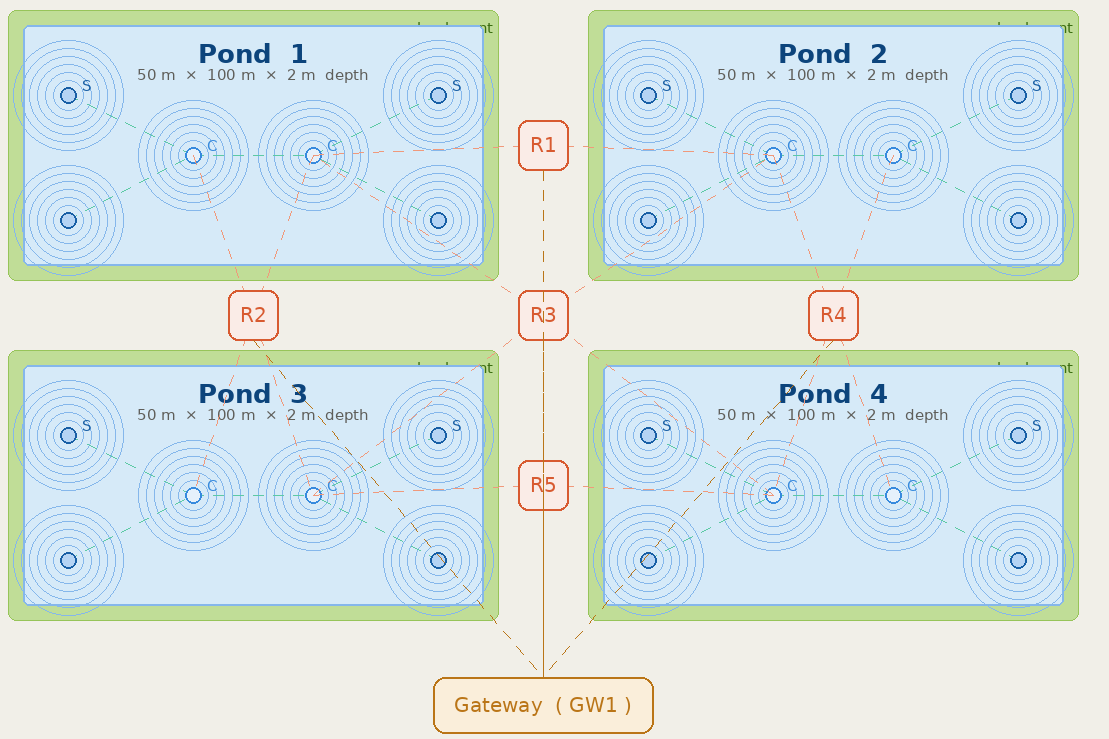


Figure A1: Coverage Map Tiers


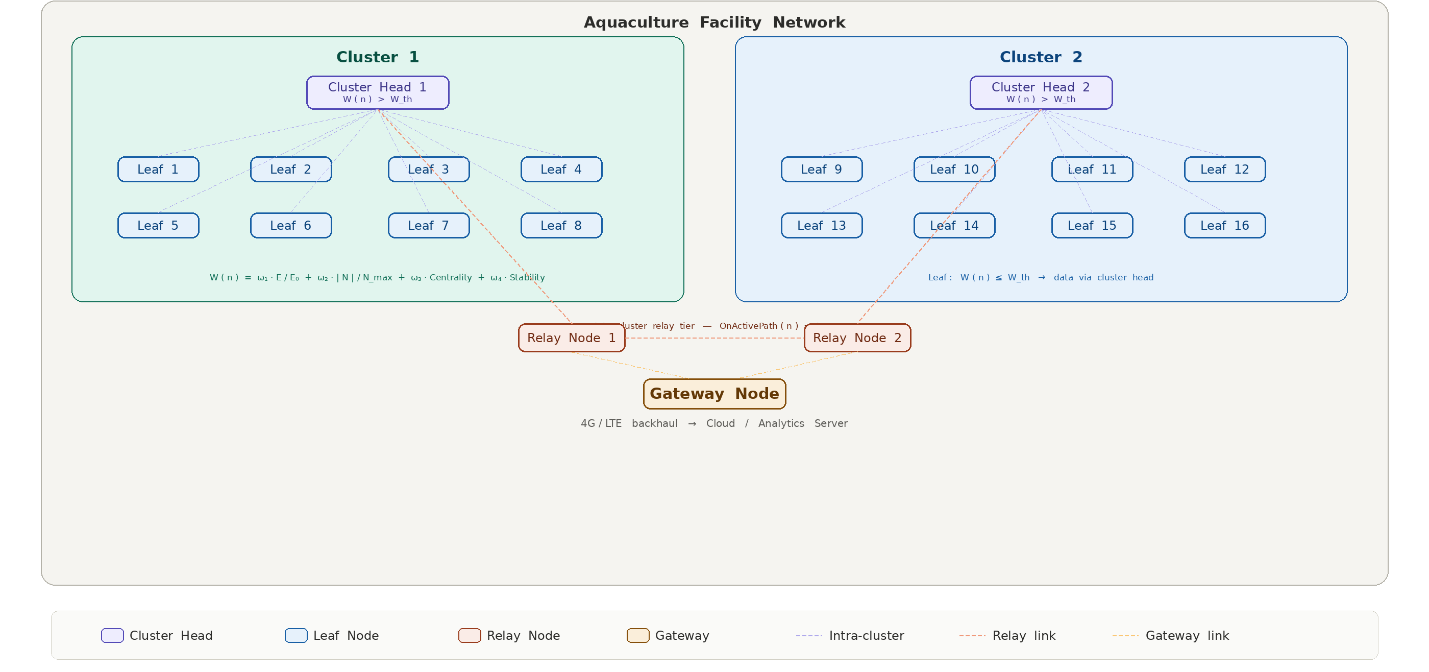


Figure A2: Agriculture Facility Network

Table S1: STP parameters

| Parameter | Base Value | Enhanced Value | Range |
| --- | --- | --- | --- |
| STP RTO (RF) | 15 ms | 12 ms | 5–50 ms |
| STP RTO (optical) | 50 ms | 40 ms | 20–100 ms |
| STP RTO (acoustic) | 200 ms | 150 ms | 100–500 ms |
| STP max retransmissions | 3 | 4 | 1–6 |
| STP duplicate window | 64 | 128 | 32–256 |

**Table S2.** Statistical significance analysis.

| Comparison | Metric | t-statistic | p-value | Cohen's d |
| --- | --- | --- | --- | --- |
| vs LoRaWAN | Latency | 28.45 | <0.001 | 2.34 |
| vs LoRaWAN | PDR | 15.67 | <0.001 | 1.28 |
| vs LoRaWAN | Throughput | 42.34 | <0.001 | 3.47 |
| vs Zigbee | Latency | 18.23 | <0.001 | 1.49 |
| vs Zigbee | Energy | 34.56 | <0.001 | 2.83 |
| vs Zigbee | Lifetime | 38.92 | <0.001 | 3.19 |
| vs Acoustic | Latency | 56.78 | <0.001 | 4.65 |
| vs BeeHive | PDR | 22.45 | <0.001 | 1.84 |
| vs AODV | PDR | 28.67 | <0.001 | 2.35 |

**Table S3.** Ablation study results.

| Configuration | Latency (ms) | PDR (%) | Throughput (Mbps) | Energy (mJ/bit) |
| --- | --- | --- | --- | --- |
| Complete | 12.3 | 99.7 | 2.4 | 0.23 |
| Without BPCM | 18.7 | 98.2 | 1.6 | 0.31 |
| Without CICA | 16.4 | 97.8 | 1.9 | 0.28 |
| Without DAGRP | 24.5 | 94.3 | 2.1 | 0.34 |
| Without TTSO | 19.8 | 96.2 | 2.2 | 0.29 |
| Without GFEB | 12.8 | 99.4 | 2.3 | 0.24 |
| Without PSP | 14.2 | 98.9 | 2.2 | 0.26 |
| Without ICCC | 15.6 | 97.4 | 2.0 | 0.27 |

**Table S4.** Economic impact analysis.

| Category | F1-Norway | F2-Egypt | F3-Thailand | F4-Greece |
| --- | --- | --- | --- | --- |
| Feed optimization (€/year) | 156,000 | 45,000 | 38,000 | 78,000 |
| Disease detection (€/year) | 124,000 | 34,000 | 52,000 | 67,000 |
| Mortality reduction (€/year) | 89,000 | 28,000 | 42,000 | 56,000 |
| Labor efficiency (€/year) | 34,000 | 12,000 | 8,000 | 18,000 |
| Equipment life (€/year) | 23,000 | 8,000 | 6,000 | 12,000 |
| Total benefit (€/year) | 426,000 | 127,000 | 146,000 | 231,000 |
| System cost (€) | 89,000 | 42,000 | 48,000 | 62,000 |
| Payback (months) | 2.5 | 4.0 | 3.9 | 3.2 |

**
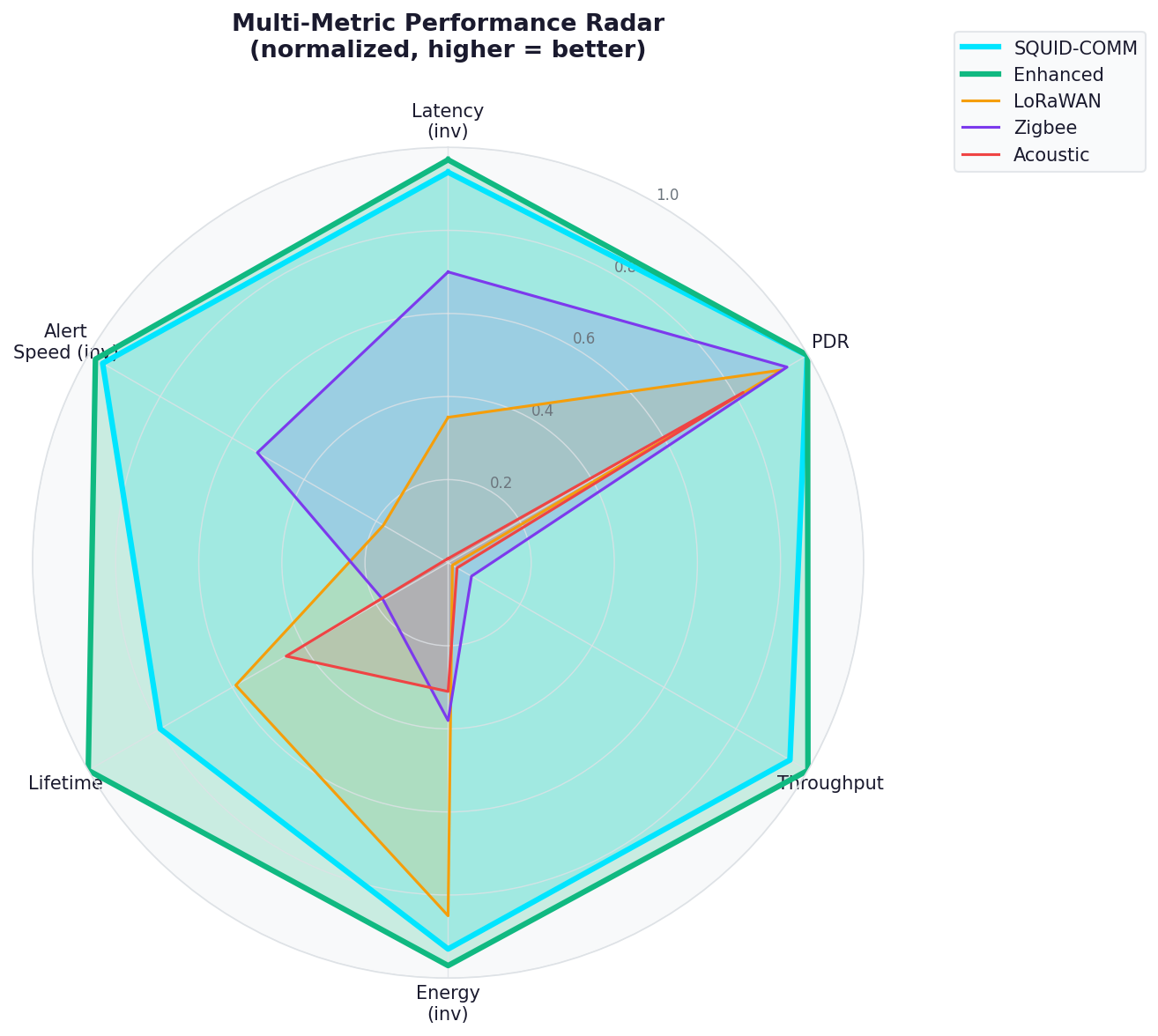
**

**Figure A3: Multi-Metric Comparison**

**
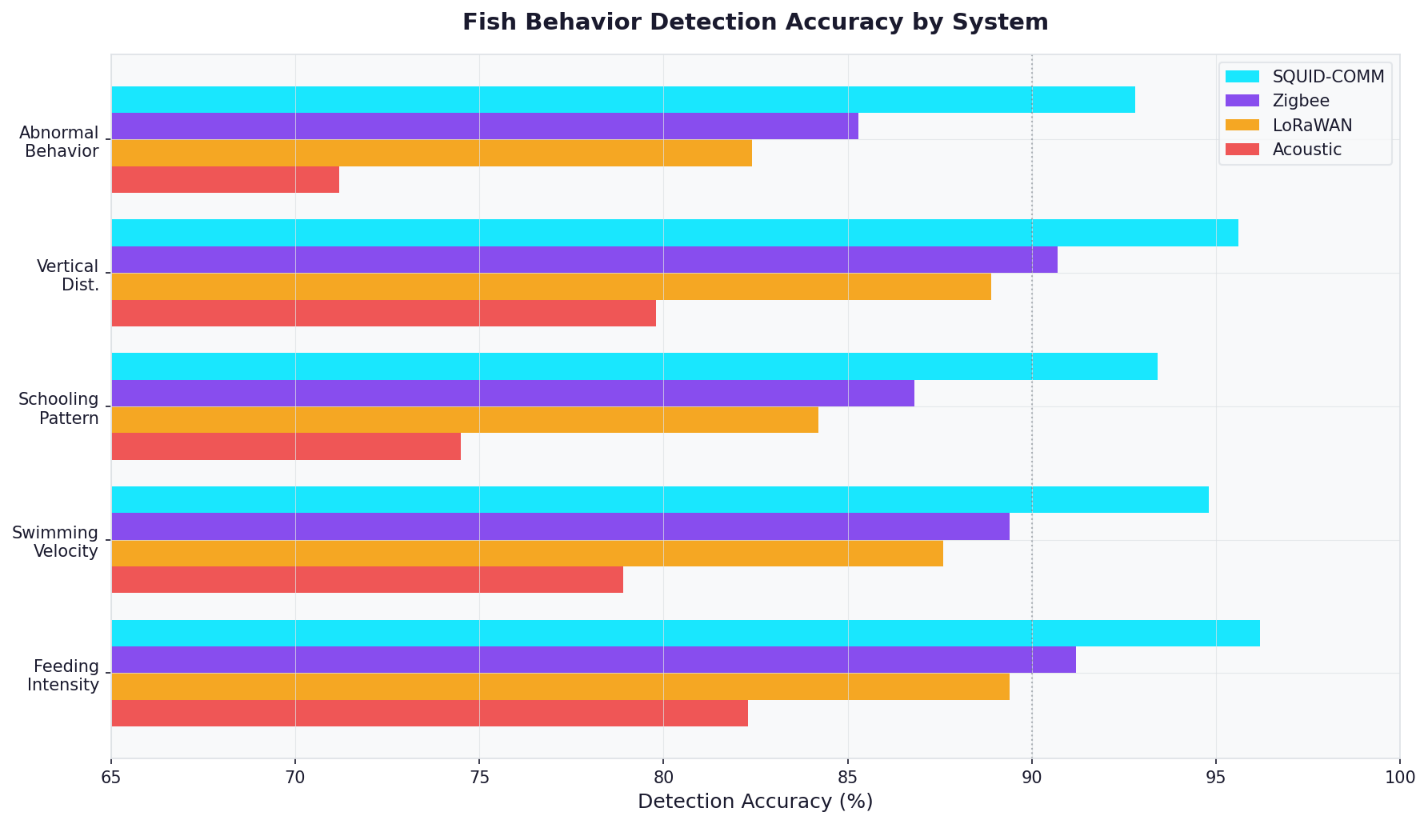
**

**Figure A4: Fish Behavior Detection**

**
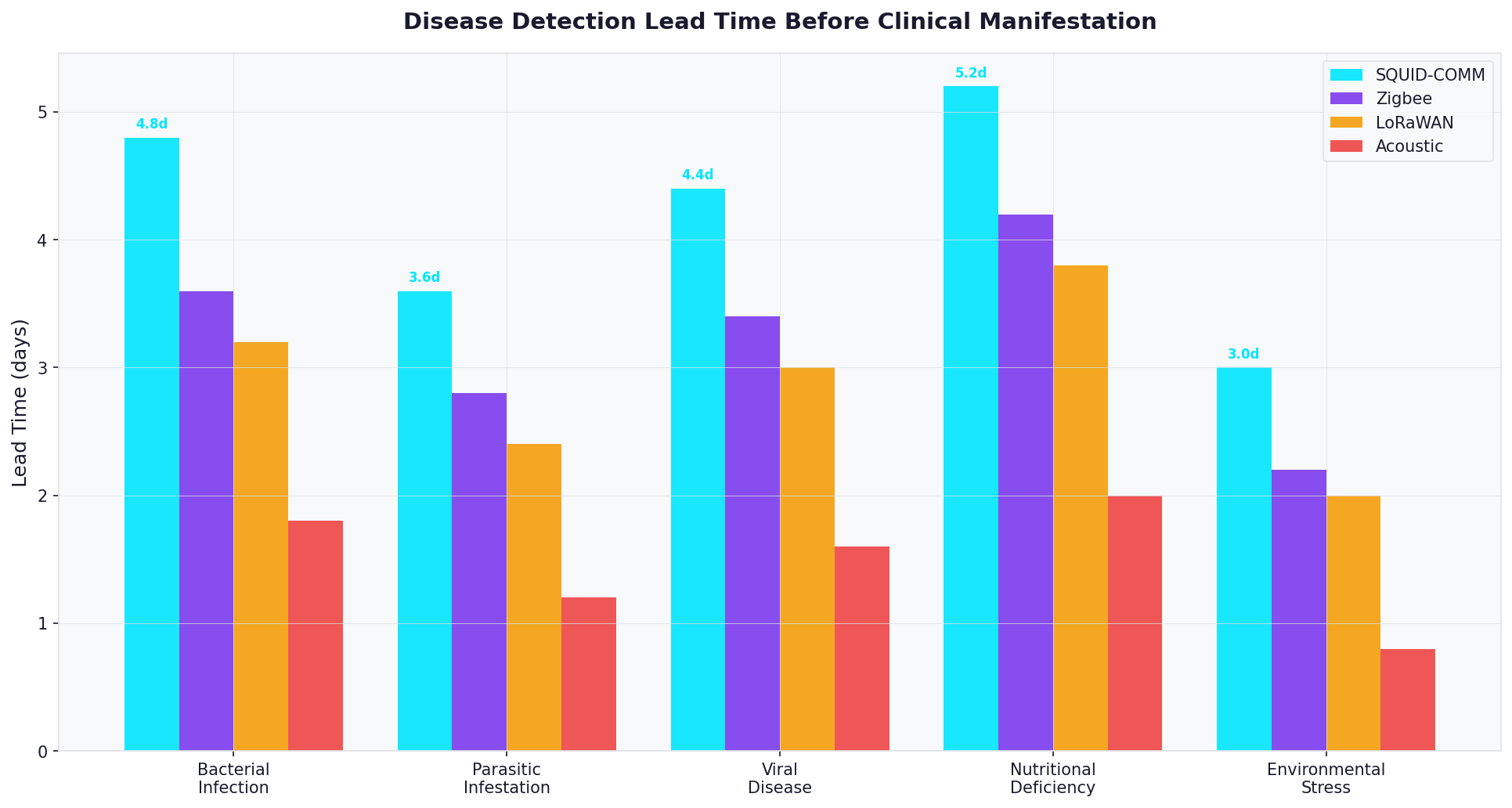
**

**Figure A5: Disease Detection Lead Time**

**
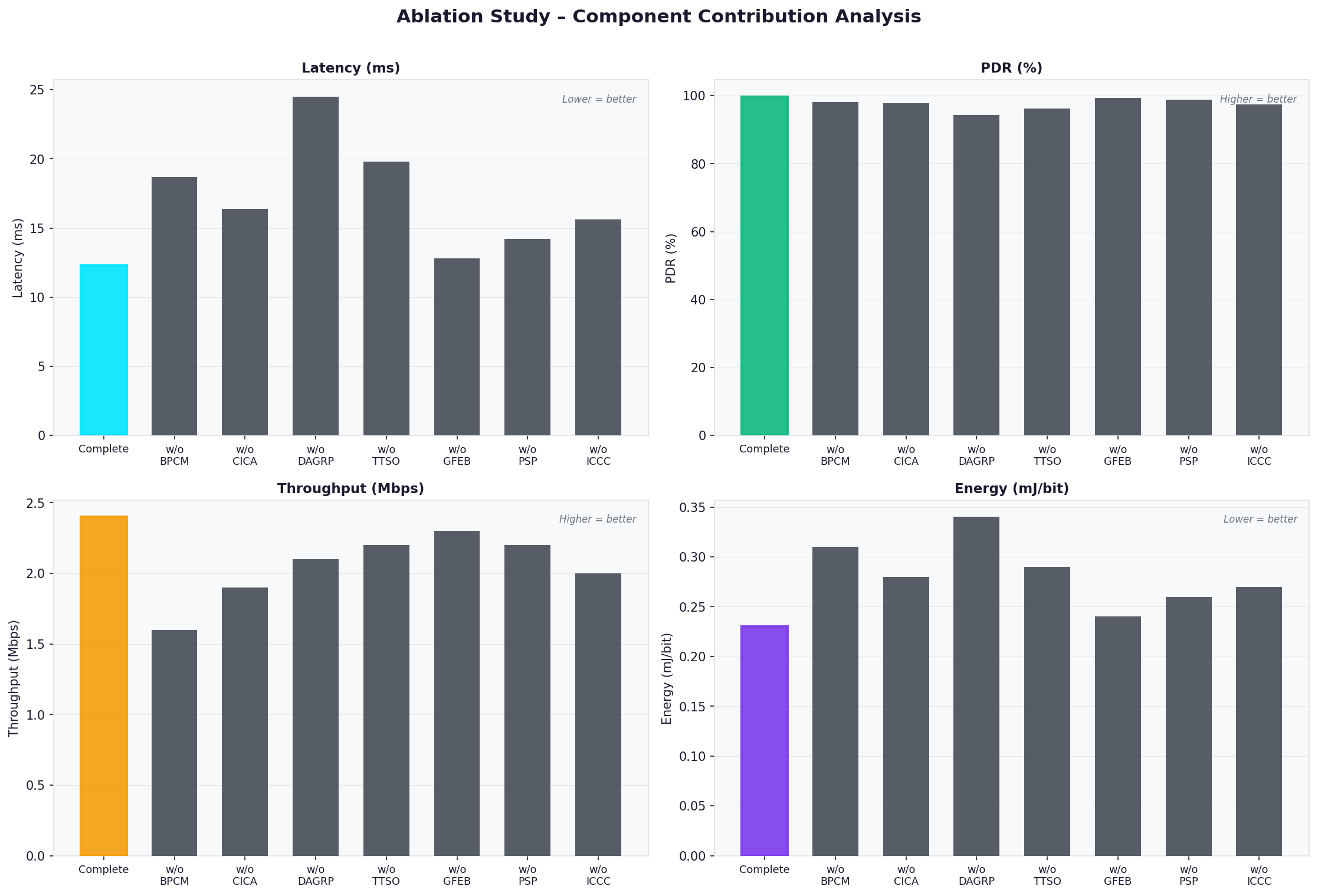
**

**Figure A6: Ablation Study**

**
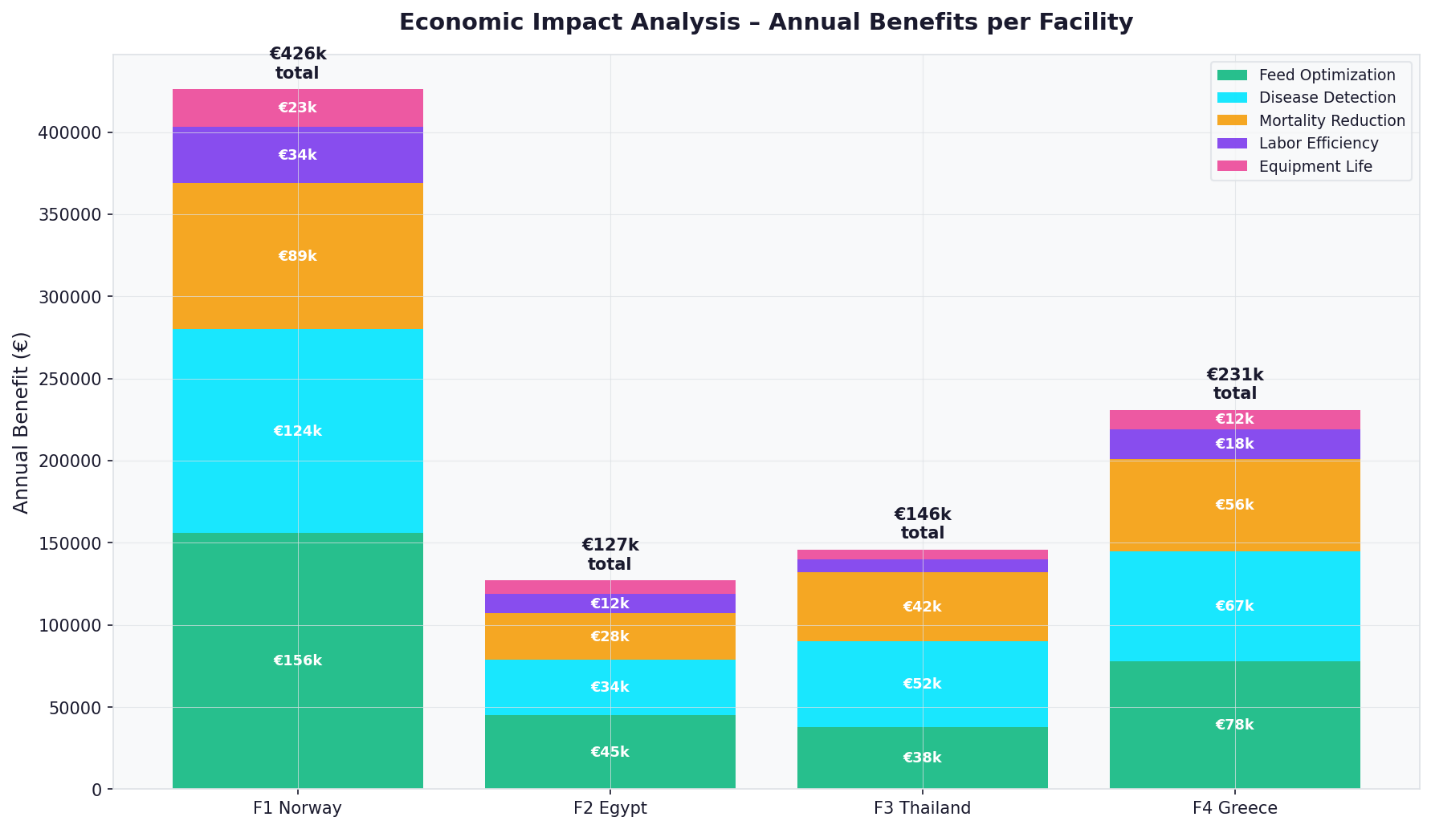
**

**Figure A7: Economic Impact**

**
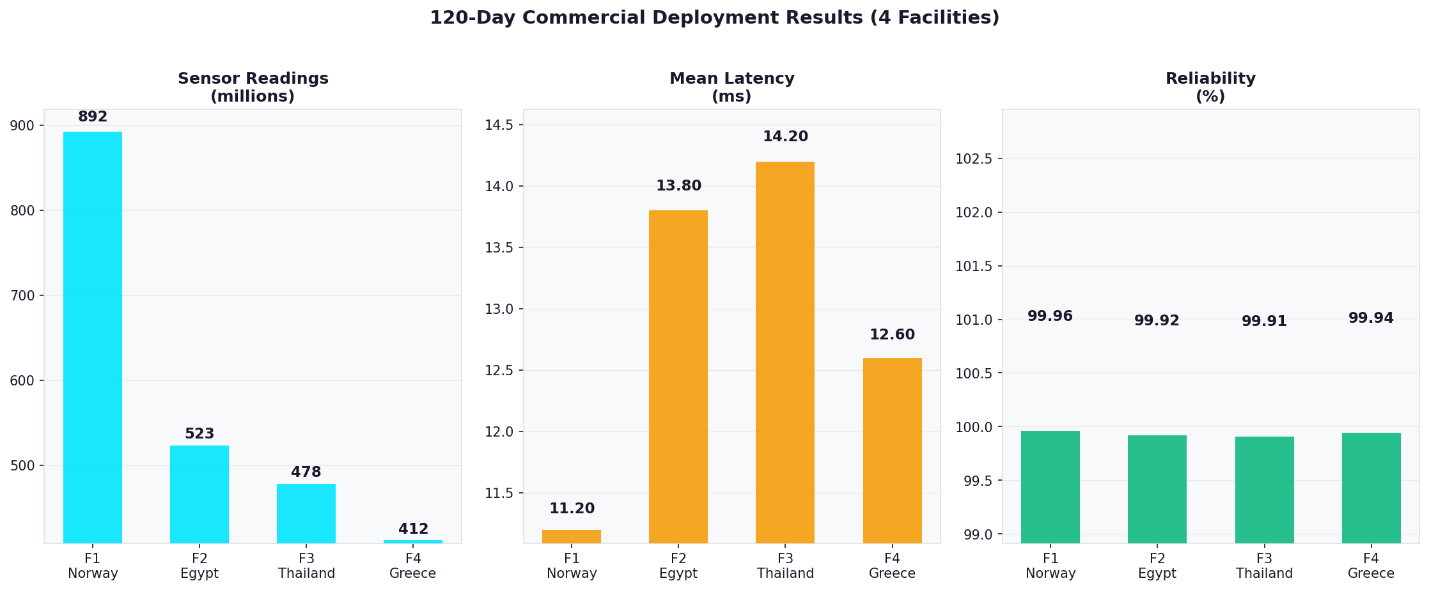
**

**Figure A8:** **Commercial Deployment**

**
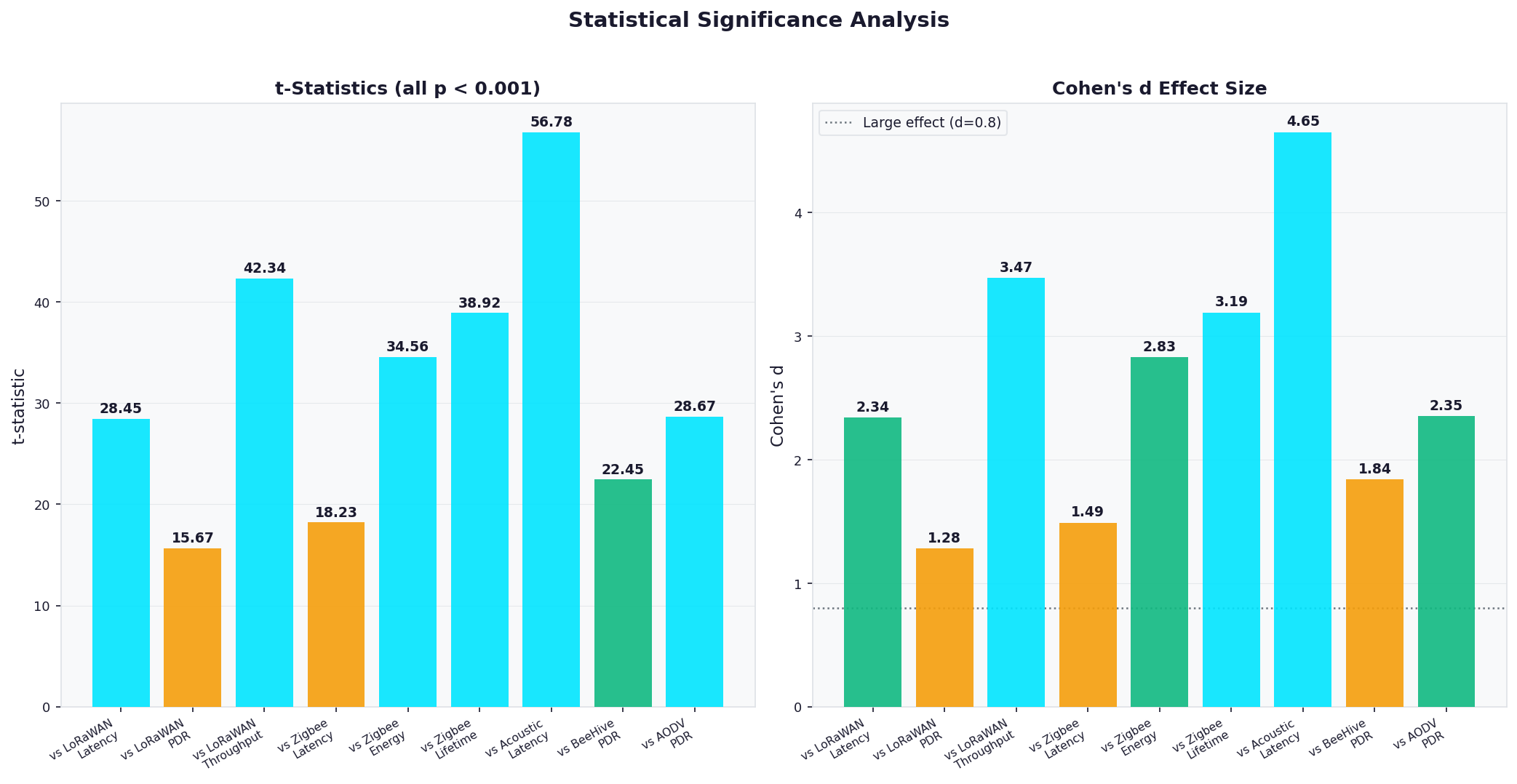
**

**Figure A9: Statistical Significance (t & Cohen's d)**
